# Measurements of C-reactive protein (CRP) and nerve-growth-factor (NGF) concentrations in serum and urine samples of dogs with neurologic disorders

**DOI:** 10.1186/s12917-015-0628-x

**Published:** 2016-01-08

**Authors:** Ulrike Kordass, Regina Carlson, Veronika Maria Stein, Andrea Tipold

**Affiliations:** Department of Small Animal Medicine and Surgery, University of Veterinary Medicine Hannover, Hannover, Germany

**Keywords:** Dog, Nerve growth factor, C-reactive protein, Micturition disorders, Spinal cord disease, Cystitis

## Abstract

**Background:**

The purpose of this study was to prove the hypothesis that C-reactive protein (CRP) and nerve growth factor (NGF) may be potential biomarkers for lower urinary tract disorders and may be able to distinguish between micturition dysfunctions of different origin in dogs with spinal cord diseases. NGF- and CRP- concentrations were measured in serum and urine samples using specific ELISA-Kits. Results in urine were standardized by urine-creatinine levels.

**Results:**

CRP in serum was detectable in 32/76 and in urine samples in 40/76 patients. NGF could be measured in all serum and in 70/76 urine samples. Urinary CRP concentrations were significantly higher in dogs with micturition dysfunction (*p* = 0.0009) and in dogs with different neurological diseases (*p* = 0.0020) compared to the control group. However, comparing dogs with spinal cord disorders with and without associated micturition dysfunction no significant difference could be detected for NGF and CRP values in urine or serum samples. Additionally, levels did not decrease significantly, when measured at the time when the dogs regained the ability to urinate properly (urinary NGF *p* = 0.7962; urinary CRP *p* = 0.078). Urine samples with bacteria and/or leukocytes had no significant increase in urinary NGF (*p* = 0.1112) or CRP (*p* = 0.0534) concentrations, but higher CRP-levels in urine from dogs with cystitis were found compared to dogs without signs of cystitis.

**Conclusions:**

From these data we conclude that neither CRP nor NGF in urine or serum can be considered as reliable biomarkers for micturition disorders in dogs with spinal cord disorders in a clinical setting, but their production might be part of the pathogenesis of such disorders. Significantly higher levels of CRP could be found in the urine of dogs with micturition dysfunctions compared to control dogs. This phenomenon could potentially be explained by unspecific extrahepatic CRP production by smooth muscle cells in the dilated bladder.

## Background

Complications with the voluntary control of micturition are commonly observed in disorders of the nervous system [1] and can worsen the prognosis in intervertebral disc diseases [2], a common neurological disorder in dogs [3]. Intervertebral disc disease cranial to the lumbosacral level can affect the voluntary voiding process [4] and might result in a detrusor-urethral dyssynergia with an areflectic bladder and urine retention [5]. Micturition disorders may be caused by the neurological disorder itself or an associated cystitis [6] precipitated by bacterial infections [7]. Escherichia coli is the most common isolate from canine urine [8, 9]. The pathogens reach the bladder from either gastrointestinal sources or the skin via the urethra [7]. The clinical signs are either pollakiurie, stranguria, dysuria, hematuria, inappropriate urination or periuria [10] and are difficult to distinguish from a neurogenic induced micturition disorder. Dogs with spinal cord injuries display a high prevalence of developing urinary tract infections [11, 12] even after recovery of voluntary urination [13].

Therefore, a biomarker helping to distinguish between neurogenic and non-neurogenic micturition disorders might be useful for pathogenesis studies and initiating appropriate treatment.

A biomarker should be an indicator for a specific disease and is supposed to identify the presence, severity, progression and the response to a specific treatment [14, 15]. Specific cells, enzymes, hormones, genes or gene products can be used as biomarkers [16]. The measurement can be performed in different tissues or body fluids [14, 16]. Two of the urinary biomarkers identified in human medicine so far are the nerve growth factor (NGF) and the C-reactive protein (CRP) [16].

NGF being a well-known neurotrophin, plays an important role in conditions of inflammatory and neuropathic pain [17, 18]. It is responsible for the growth, survival and development of neural cells, as well as the regulation of neural plasticity in the micturition pathway [19]. NGF is produced by urethral cells and cells of the detrusor muscle [20]. Different studies show that NGF is involved in the development of neurogenic bladder overactivity at the spinal level following the disruption of supraspinal micturition control after spinal cord injury [19]. Raised NGF levels in serum could be found in bladder dysfunctions [21]. There are several studies in humans on NGF as a urinary biomarker [22] showing increased NGF levels in patients with urinary tract disorders [21, 23, 24].

CRP is an acute-phase protein, mainly produced in the liver and elevated after infection, inflammation or trauma. The CRP concentration rises depending on the tissue damage [25]. In human studies CRP in serum is increased in patients with lower urinary tract disorders and it correlates with the severity of clinical signs [26, 27]. In dogs, CRP in serum can be used as an indicator of inflammatory response to experimentally induced cystitis [28].

Therefore, we hypothesize that CRP and NGF are measurable in serum and urine samples and can be used as biomarkers in either serum or urine to distinguish between micturition disorders caused by neurologic dysfunctions or bacterial cystitis in dogs with spinal cord disease.

## Results

76 dogs were included in the study. The patients were categorized into four different groups (Table 1).

**Table 1:** Categorization of the dogs into four different groups

	Groups	N
1	Different neurological disorders (ND)	14
2	Spinal cord disorders without micturition problems (SC)	15
3	Spinal cord disorders with micturition problems (SC MD)	27
4	Healthy dogs (control)	20

Diseases included in the study are summarized in Table 2. Urinary CRP could not be detected in each patient. The highest levels were found in dogs with spinal cord disorders and micturition problems. 80 % of the dogs in the control group, 64 % with different neurological disorders, 41 % with spinal cord disorders with micturition problems and 29 % with spinal cord disorders without micturition problems had detectable CRP values. In detail, urinary CRP was measurable in 16/20 dogs of the control group with a mean concentration of 1.08 ng/ml (range: 0–1.76 ng/ml). After normalizing CRP to creatinine the mean ratio was 0.01 CRP/Crea (range: 0–0.02 CRP/Crea) (Table 3). In 9/14 of the patients within the group of different neurological disorders urinary CRP was measurable with a mean concentration of 2.21 ng/ml (range: 0–11.39 ng/ml) and after normalizing to creatinine the mean ratio accounted for 0.04 CRP/Crea (range: 0–0.26 CRP/Crea) (Table 3). In the group “spinal cord disorders without micturition problems” CRP was measurable in 4/15 dogs with a mean concentration of 1.45 ng/ml (range: 0–14.91 ng/ml) and after normalizing to creatinine the mean ratio was 0.02 CRP/Crea (0–0.17 CRP/Crea) (Table 3). CRP was measurable in 11/27 patients of the group with the spinal cord disorders with micturition problems with a mean concentration of 9.22 ng/ml (0–131 ng/ml) and after normalizing to creatinine a mean ration of 0.12 CRP/Crea (0–1.14 CRP/Crea) was detected (Table 3).

**Table 2:** Different diseases included in the study

Disease	N	ND	SC	SCMD
Idiopathic epilepsy	5	5		
Cryptogenic epilepsy	1	1		
Meningoencephalitis	3	3		
Steroid-responsive meningitis-arteritis	1	1		
Vestibular disease	2	2		
Neoplasia intracranial	1	1		
Polyneuropathy	1	1		
Intervertebral disc herniation	39		15	24
Myelitis	1			1
Vertebral dislocation	2			2
Total	56			

Distribution of the diseases in the different groups (*N* = number): neurological disorders (ND), spinal cord disorders without micturition dysfunction (SC), spinal cord disorders with micturition dysfunction (SCMD)

**Table 3:** C-reactive protein and nerve-growth-factor levels in urine and serum samples

	CRP urine ng/ml	CRP/Crea urine	CRP serum ng/ml	NGF urine	NGF/Crea urine	NGF serum pg/ml
Control	1.08 (0–1.76)	0.01 (0–0.02)	946 (0–6820)	76.60 (7.17-250)	0.50 (0.06-3.01)	179 (104–250)
Neurological Disorders	2.21 (0–11.39)	0.04 (0–0.26)	22452 (0–208658)	31.98 (0–150)	0.61 (0–2.41)	164 (40–250)
SC	01.45 (0–14.91)	0.02 (0–0.1721)	14764 (0–92035)	63.24 (0–198)	1.00 (0–3.43)	166 (24.77-250)
SC MD	9.22 (0–131)	0.12 (0–1.1462)	9235 (0–81719)	58.11 (0–250)	0.73 (0–4.40)	167 (46–250)

C-reactive protein (CRP) and nerve-growth-factor (NGF) levels in urine and serum samples (mean, range in brackets), CRP/Creatinine and NGF/Creatinine ratios in the different groups: control group (*n* = 20), neurological disorders (*n* = 14), spinal cord disorders without micturition dysfunctions (SC) (*n* = 15) and spinal cord disorders with micturition dysfunctions (SC MP) (*n* = 27)

Serum CRP could be detected in 4/20 patients in the control group with a mean of 946 ng/ml (range: 0–6820 ng/ml) (Table 3). The mean level in 7/14 patients with neurological disorders was 22,452 ng/ml (range: 0–208,658 ng/ml) (Table 3). The wide range of the values was caused by including dogs with inflammatory CNS diseases. One dog suffering from steroid-responsive meningitis-arteritis displayed the highest value (208,658 ng/ml). This sample was additionally used as a positive control [29] to evaluate the ELISA test, since CRP was not measurable in all serum samples. In 9/15 dogs with spinal cord disorders without micturition problems CRP was measurable with a mean concentration of 14,764 ng/ml (range: 0–92,035 ng/ml) and in 12/27 dogs with micturition problems a mean concentration of 9235 ng/ml (range 0–81,719 ng/ml) could be detected (Table 3).

In all of the 20 patients in the control group urinary NGF was measurable and ranged between 7.17-250 pg/ml (mean 76.60 pg/ml). After normalizing to creatinine a mean ratio of 0.50 NGF/Crea (range 0.06 - 3.00 NGF/Crea) could be found (Table 3). In 13/14 patients with different neurological disorders NGF was measurable with a mean concentration of 31.98 pg/ml (range 0–150 pg/ml) and after normalizing to creatinine the mean ratio was 0.61 NGF/Crea (range 0–2.41 NGF/Crea) (Table 3). Urinary NGF mean value was 63.24 pg/ml (range 0–198 pg/ml) in 13/15 patients with spinal cord disorders without micturition problems (Table 3), the mean NGF/Creatinine ratio was 1.00 NGF/Crea (range 0–3.43 NGF/Crea) (Table 3). In 24/27 dogs with micturition problems the mean concentration of NGF was 58.11 pg/ml (range 0–250 pg/ml). The range of the ratio after normalizing to creatinine was 0–4.40 NGF/Crea with a mean of 0.73 NGF/Crea (Table 3).

Serum NGF was detectable in all of the analyzed serum samples. The mean concentration of the control group was 179 pg/ml (range: 104–250 pg/ml), of different neurological disorders 164 pg/ml (range: 40–250 pg/ml), of the spinal cord disorders without micturition problems 166 pg/ml (range: 24.77-250 pg/ml) and the spinal cord disorders with micturition problems showed a mean concentration of 167 pg/ml (range: 46–250 pg/ml) (Table 3).

In the first group including all dogs, significant differences measuring the CRP/Crea-ratio could be found between the spinal cord disorders without micturition problems and the control group (*p* = 0.0118) as well as with different neurological disorders (*p* = 0.0445). No further significant differences could be detected.

The CRP levels in serum were significantly different in the control group and the spinal cord disorders without micturition problems (*p* = 0.0181) (Table 4), no further significant differences could be evaluated (Table 4).

**Table 4:** *p*-values of the different concentrations of C-reactive protein and nerve growth factor in serum and urine samples

	CRP/Crea Urine	CRP/Crea Urine all	CRP Serum	NGF/Crea Urine	NGF Serum
Control Group vs Neurological Disorders	0.0020*	0.2510	0.0670	0.6119	0.6450
Control Group vs SC	0.5391	0.0118*	0.0181*	0.4334	0.9591
Control Group vs SC with micturition dysfunction	0.0009*	0.4010	0.1113	0.8546	0.6484
Neurological Disorders vs SC without micturition dysfunction	0.6997	0.0445*	0.4503	0.4712	0.7315
Neurological Disorders vs SC with micturition dysfunction	0.4033	0.3369	0.8576	0.7207	0.9778
SC without micturition dysfunction vs SC with micturition dysfunction	0.3961	0.2837	0.3401	0.4620	0.8298
Cystitis vs No cystits		0.0534		0.1112	
Dogs unable to urinate vs Dogs with normal voiding		0.0778		0.7962	

*P*-values of the different concentrations of C-reactive protein (CRP) and nerve growth factor (NGF) in serum and after normalizing to creatinine (CRP/ Crea; NGF/Crea) in urine samples for the different groups. Measurements including all CRP/Crea values are shown in the column “CRP/Crea urine all”. Calculations of samples only with detectable values are shown in the column “CRP/Crea Urine”. *P*-values comparing dogs with spinal cord disorders and associated micturition dysfunction with (cystitis) and without cystitis (no cystitis) and dogs regaining the ability to urinate (dogs with normal voiding) are shown in the last rows. Significances are highlighted with an asterisk (*).Spinal Cord disorders (SC)

**Fig. 1:**
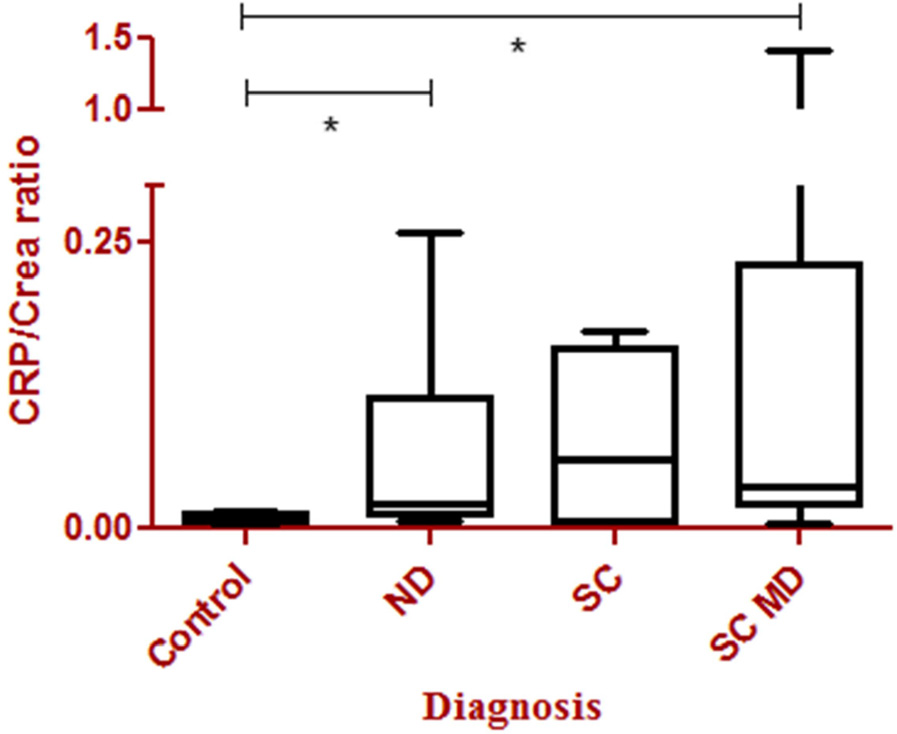
C-reactive protein/ creatinine ratios in urine. C-reactive protein/creatinine (CRP/Crea) concentrations: significant differences (*) between the control group (healthy dogs *n* = 20) and different neurological disorders (*n* = 14) (ND) (*p* = 0.0020) as well as spinal cord disorders with micturition dysfunction (*n* = 15) (SC MD) (*p* = 0.0009). The boxplots are indicating minimums and maximums, lower and upper quartiles, and medians

Additionally, we determined the significances between the concentrations of CRP/Crea in the different groups in the urine only within measurable samples (Fig. 1 and Table 4). Significant differences could be detected between the healthy control group and the neurological disorders (*p* = 0.0020) as well as the spinal cord disorders with micturition problems (*p* = 0.0009). No significant difference could be found between spinal cord disorders with and without micturition problems (*p* = 0.3961). However, the highest values of CRP/Crea-ratios occurred in dogs with micturition problems.

**Fig. 2:**
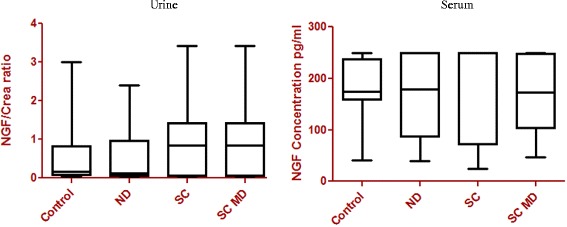
Ratios of nerve growth factor/ creatinine in urine and nerve growth factor levels in serum in the different groups. Ratios of nerve growth factor/creatinine in urine (NGF/Crea) and nerve growth factor levels in serum (NGF Serum) in the different groups: control group (healthy dogs; *n* = 20), neurological disorders (ND; *n* = 14), spinal cord disorders without micturition dysfunctions (SC; *n* = 15) and spinal cord disorders with micturition dysfunction (SC MD; *n* = 27). No significant differences could be detected. The boxplots are indicating minimums and maximums, lower and upper quartiles and medians

No significant differences could be detected in the urine of NGF/Crea ratios and the NGF levels in serum in the four different groups (Fig. 2 and Table 4).

Neither urinary NGF/Crea (*p* = 0.7962) nor CRP/Crea (*p* = 0.0778) showed significantly decreased levels after dogs with micturition disorders regained the ability to urinate (Table 4).

**Fig. 3:**
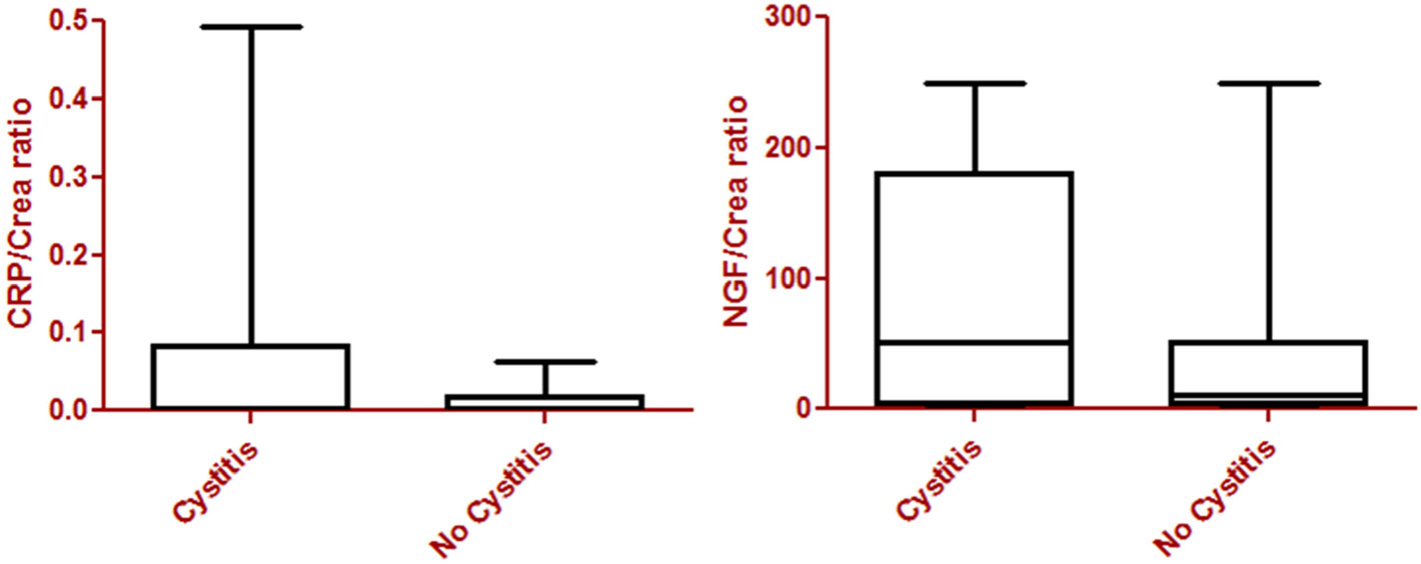
Spinal cord disorders with micturition dysfunction. Values of C-reactive protein/creatinine ratio (CRP/Crea) and nerve-growth factor/ creatinine ratio (NGF/Crea) of dogs with spinal cord disorders with micturition dysfunction (*n* = 27). No significant difference in CRP/Crea (*p* = 0.0534) or NGF/Crea (*p* = 0.1112) could be detected within the group of dogs with micturition dysfunctions with (*n* = 10) or without cystitis (*n* = 17). However, higher concentrations could be found in dogs with cystitis in the NGF and CRP measurements. The boxplots are indicating minimums and maximums, lower and upper quartiles, and medians

The urine examination revealed leukocytes (8/10) or bacteria and leukocytes (2/10) in 10 samples of the 27 dogs with micturition dysfunction. CRP/Crea and NGF/Crea values of dogs with cystitis were higher than in dogs without bacteria and/or leukocytes in the urine. However, this increase was not statistically significant (NGF/Crea *p* = 0.1112 and CRP/Crea *p* = 0.0534) (Fig. 3 and Table 4).

## Discussion

In the current study the hypothesis had to be proven, that CRP or NGF are measurable in urine and serum samples of dogs and that these two proteins may be involved in the pathogenesis of micturition disorders. The aim of the study was to establish CRP and NGF as biomarkers helping to distinguish between neurogenic and non-neurogenic micturition disorders. In this study we concentrated on micturition disorders caused by diseases of the spinal cord and associated bacterial cystitis. Indeed, CRP and NGF are measurable with specific, commercially available ELISA kits in both, urine and serum of dogs as described before [24, 30]. Urine samples were compared to creatinine values of the urine to overcome the different dilutions between the samples [24].

However, the second part of the hypothesis could not be proven. No differences of NGF in serum or urine could be found between the different groups of neurological disorders and the control group.

NGF levels can rise in many different conditions such as neurologic diseases, endocrinological disorders and diseases of the immune system [31]. It is produced by many different cells, including urothelial cells, smooth muscle cells and mast cells [21]. Urinary NGF in particular was examined in lower urinary tract disorders [23]. HT Liu and HC Kuo [21] demonstrated that serum and urine NGF levels were increased in humans suffering from interstitial cystitis and bladder pain syndrome, but no correlation could be found between serum and urinary NGF and the clinical signs. The authors suggest that other systemic disorders are also involved and influence the NGF-levels, especially the levels in serum [21]. In the current study, we tried to rule out other systemic diseases by routine blood examination and different imaging techniques such as ultrasound and radiographs of abdomen and thorax to exclude such an influence.

Urinary NGF levels were also found to be increased in cerebrovascular events and correlate with the severity of neurological impairment but not with urinary tract dysfunctions [32]. In our study dogs with mild and severe spinal cord injury were included leading most probably to the high range of the values and overlap between the groups.

The detected high variability of NGF-levels could be explained by comparing with the following studies. A study with rats experiencing stress showed increased NGF-values in serum [17]. In the current study, dogs with neurological dysfunctions had to be examined in the clinic. It could be expected that these dogs were suffering from stress because of their disease and the medical intervention, which could interfere with the production of NGF.

In human reports urine has been collected, when the bladder was full and the patients had a strong desire to void. NGF in urine increases physiologically while bladder stretching in healthy people [33]. In dogs it is difficult to standardize the urine collection according to those factors. However, urine samples were always taken from a filled bladder either through spontaneous voiding (walking the dog only when the bladder was filled), manual expression or cystocentesis to overcome the influence of heterogenous bladder stretching as much as possible.

NGF in urinary bladder biopsies of rats with spinal cord injuries was found to decrease four days to four weeks after injury, whereas the amount of NGF increased five to six weeks after the injury [34]. The mRNA-levels of total urinary bladder NGF decreased significantly in acute or chronic cyclophosphamide-induced cystitis compared to control animals. In the current study urine and serum collection was performed, when the dogs were presented in the clinic, therefore standardization according to the onset of the clinical signs was not feasible. The dogs had been suffering from neurological signs and/or micturition disorder for a variable amount of time. This well-known shortcoming of clinical studies could be a reason for measuring a wide range of NGF-values.

In conclusion, NGF cannot be developed to serve as a specific biomarker for severity of micturition disorders in a clinical setting shown by the results of the current study and the fact that NGF secretion in urine and especially serum can be influenced by many different factors.

CRP is an acute-phase protein, which is mainly produced by the liver and is raised in conditions of inflammation and tissue damage [25].

In a study performed by Chuang et al. (2010) the authors demonstrated higher serum levels in humans with lower urinary tract disorders (overactive bladder syndrome), but urinary CRP and mRNA expression of CRP in bladder biopsies were barely detectable. Detection of elevated urinary CRP has been reported before in dogs with renal disease caused by leishmaniasis [35], babesiosis [36] and pyometra [37]. A time-resolved immunofluorimetic assay was used to determine the urinary CRP concentration in dogs with leishmaniasis [35], whereas urine samples of dogs with babesiosis and pyometra were analyzed using an ELISA-system as in the current study [36] .

In our study CRP could not be detected in each urine sample, even the study design, control probes and the above mentioned measurements are proof that CRP is measurable in urine of dogs using an ELISA. Evaluating detectable urine samples only a significant higher concentration of CRP/Crea-levels could be found comparing the control group and different neurological disorders and the control group with spinal cord disorders with micturition problems. Measurements in the group spinal cord disorders without micturition problems displayed the widest range. In this group the severity of neurological/motor dysfunction varied the most. Additionally, CRP/Crea levels in dogs suffering from spinal cord disorders with micturition dysfunction with cystitis were higher than in dogs without cystitis. In a study done by KW Seo, JB Lee, JO Ahn, HW Lee, CY Hwang, HY Youn and CW Lee [28], dogs with experimentally induced cystitis showed higher CRP values in serum than the control group. Since no significances could be detected in dogs with spinal cord disorders with and without cystitis, a comparison with a control group of dogs without neurological dysfunction and cystitis was abandoned. Such dogs suffered also from systemic diseases, which would have influenced additionally CRP and NGF concentrations (data not shown).

It is anticipated that many tissues of the body can produce CRP as a part of their own immune system defense [38]. Extrahepatic syntheses have been reported in neuron, atherosclerotic plaques, monocytes and lymphocytes. The mechanisms underlying the extrahepatic synthesis are unknown [38, 39]. In humans CRP can be produced by coronary artery smooth muscle cells [40]. Therefore, a production of CRP by smooth muscle cells of the bladder might be involved in the pathogenesis of micturition disorders.

The highest concentrations can be measured two to four days after the onset of the disease [41]. The individual CRP concentrations can vary significantly between different dogs [42]. S Yamamoto, T Shida, S Miyaji, H Santsuka, H Fujise, K Mukawa, E Furukawa, T Nagae and M Naiki [41] reported increased and decreased CRP serum values in dogs with different disorders such as enteritis, eye diseases and nephritis. Some of the dogs whose medical condition had improved after the treatment had increased, others decreased CRP levels [41]. Levels of CRP in serum can increase after surgery. Moreover, the degree to which the levels rise, corresponds to the surgical trauma [43]. In our study some patients underwent surgical treatment and others did not. This could be a reason for different serum CRP values specifically the high range of the CRP levels. However, a diagnostic biomarker for use in a clinical setting should overcome such differences in treatment of individual patients with spinal cord disorders.

## Conclusions

In conclusion, CRP and NGF are measureable in both urine and serum of dogs. Levels of CRP in the urine of dogs with spinal cord disorders with micturition problems were elevated. However, not all dogs with micturition disorders had detectable CRP values. No significant differences of CRP and NGF in serum or urine in the diseased groups could be detected. Therefore, it cannot be recommended to develop CRP and NGF as reliable biomarkers in micturition disorders in a clinical setting. However, these proteins could be valuable for pathogenesis studies or for control examinations of single patients in treatment studies.

## Methods

### Study design and animals

Paired urine and serum samples were collected from 76 dogs with different neurological disorders and from control dogs at the Department of Small Animal Medicine and Surgery, University of Veterinary Medicine Hannover, Germany. The study was performed according to the ethical rules of the Lower Saxony State Office for Consumer Protection and Food Safety, Germany, animal experiment number: 33.4-42502-04-13A374. An owners’ permission was granted for all dogs. The following breeds were included: mixed breed (*n* = 17), Dachshund (*n* = 10), Beagle (*n* = 8), Bernese Mountain Dog (*n* = 6), Labrador (*n* = 5), French bulldog (*n* = 4), Golden Retriever (*n* = 3), Jack Russell Terrier (*n* = 2), Shepherd (*n* = 3), Giant Schnauzer (*n* = 2), Small Münsterländer (*n* = 1), Boxer (*n* = 1), Bolonka Zwetna (*n* = 1), Miniature Schnauzer (*n* = 1), Weimaraner (*n* = 1), Saint Bernard dog (*n* = 1), Old English Sheepdog (*n* = 1), Swiss Mountain Dog (*n* = 1), Pekingese dog (*n* = 1), Chihuahua (*n* = 1), Shi Tzu (*n* = 1), Bracke (*n* = 1), German wirehaired pointer (*n* = 1). The mean age of the dogs was 5.6 years (+/− 2.8 years) and the average weight was 19.8 kg (+/−13 kg). 29 dogs were male, 21 castrated-male, 7 female and 19 spayed female.

Paired urine and serum samples were collected from patients with different neurological disorders and from the control dogs. The clinical diagnosis was established using a combination of clinical examination, neurological examination, blood and urine analysis, magnetic resonance imaging (52/76) and evaluation of cerebrospinal fluid samples (48/72). In all dogs regular clinical and laboratory control examinations were performed to rule out concurrent diseases.

The patients were categorized in 4 different groups (Table 1): healthy dogs (*n* = 20); different neurological disorders except spinal cord disorders (Table 2) (*n* = 14); spinal cord disorders with normal micturition (*n* = 15); spinal cord disorders with micturition problems (*n* = 27) (Table 5); [44] out of the 27 dogs with micturition problems, 10 urine samples were taken in a follow up study, when the dogs regained the ability to void. “Normal” micturition was assumed, when dogs with spinal cord disorders had spontaneous voiding when the bladder was filled and they could walk outdoors. Dogs were considered to be healthy, when clinical and neurological examinations were normal and no abnormalities in blood and urine analysis were observed.

**Table 5:** Number (N) of dogs with different severity of spinal cord dysfunction (grading according to Sharp and Wheeler 2005)

	N
Grade I	2
Grade II	6
Grade III	7
Grade IV	11
Grade V	16

### Sample collection

Urine samples were collected after spontaneous voiding, manual expression or cystocentesis and immediately analyzed. All urine samples of dogs with normal micturition were taken after spontaneous voiding, urine samples of dogs with micturition disorders were taken after manual expression (n = 12) or cystocentesis (*n* = 15). To rule out or to diagnose cystitis all urine samples were analyzed with a colorimetric urine analysis reagent test stripe (Urine stripes, Roche Diagnostics GmbH, Mannheim, Germany). Samples with leukocytes and/or bacteria in urine sediment analysis were considered dogs with cystitis. The specific gravity was measured with a refractometer (Manual Handrefractometer, A.KRÜSS Optronic GmbH, Hamburg, Germany). The urine creatinine level was measured to normalize CRP and NGF levels using Cobas C311 Analyzer (Roche Diagnostics GmbH, Mannheim, Germany). Urine and serum samples were centrifuged at 1000 rpm for 10 min and frozen at −20 °C until analyzed.

### Analysis of NGF

Urinary and serum NGF concentrations were determined using an immunosorbent assay system (NGF EMAX ®ImmunoAssay System- ELISA Test Kit, Madison, WI, USA). This assay was evaluated for the use of canine samples [24, 30]. The assay had a minimum sensitivity of 7.8 pg/ml, values above 250 pg/ml cannot be quantified and was performed according to the manufacturer’s instructions. Serum samples were diluted 1:50, urine samples were not diluted. Values that were above 250 pg/ml were counted as 250 pg/ml, values lower than the detection range as zero. All samples were run in duplicates and the mean of these two values was used for further evaluation.

### Analysis of CRP

CRP levels in urinary and serum samples were measured using a commercially available dog specific immunosorbent assay system (dog high- Sensitive CRP ELISA Kit, Kamiya Medical Company, Seattle, WA, USA), which can be applied for several biological fluids such as urine. The detection range was between 3,125 ng/ml and 200 ng/ml. The assays were conducted according to the manufactures instructions. The serum samples were diluted 1:1000, urine samples were diluted 1:2. Results below the detection limit were counted as zero. None of the samples were above the detection limit. All samples were run in duplicates and the mean of these two values was used for further evaluation.

### Statistical analysis

After testing for normal distribution, the Wilcoxon-Mann-Whitney-Test was used to evaluate potential significant differences between the four different groups. To distinguish between healthy dogs and dogs with cystitis a *t*-test was performed. In order to differentiate between dogs with abnormal micturition and dogs that regained ability of normal micturition a f-test was performed. To analyze the number of samples with detectable CRP and NGF values a Fisher-Yate was used. All data analyses were conducted with the statistic program package SAS®, version 9.2 (SAS Institute, Cary, NC, USA). For all statistical tests an error probability of *p* < 0.05 as significance level was chosen. Statistical calculations were performed twice: using all data including the patients without measurable CRP values (counted as zero) and a calculation including only patients with measurable values. Data of calculated *p*-values are summarized in (Table 4).

## Abbreviations

Crea: Creatinin
CRP: C-Reactive Protein
CRP/Crea: C-Reactive Protein/ Creatinin Ratio
ELISA: Enzyme Linked Immunosorbent Assay
ND: Neurological Disorders
NGF: Nerve Growth Factor
NGF/Crea: Nerve Growth Factor/ Creatinin Ratio
SC: Spinal cord disorders
SC MD: Spinal Cord disorders with micturition dysfunction
